# Neural Basis of Action Observation and Understanding From First- and Third-Person Perspectives: An fMRI Study

**DOI:** 10.3389/fnbeh.2018.00283

**Published:** 2018-11-22

**Authors:** Sheng Ge, Hui Liu, Pan Lin, Junfeng Gao, Chaoyong Xiao, Zonghong Li

**Affiliations:** ^1^Key Laboratory of Child Development and Learning Science of Ministry of Education, Research Center for Learning Science, Southeast University, Nanjing, China; ^2^Key Laboratory of Cognitive Science, College of Biomedical Engineering, South-Central University for Nationalities, Wuhan, China; ^3^Department of Radiology, The Affiliated Nanjing Brain Hospital of Nanjing Medical University, Nanjing, China

**Keywords:** action observation, action understanding, mirror neuron system, hand-object interaction, fMRI

## Abstract

Understanding the intentions of others while observing their actions is a fundamental aspect of social behavior. However, the differences in neural and functional mechanisms between observing actions from the first-person perspective (1PP) and third-person perspective (3PP) are poorly understood. The present study had two aims: (1) to delineate the neural basis of action observation and understanding from the 1PP and 3PP; and (2) to identify whether there are different activation patterns during action observation and understanding from 1PP and 3PP. We used a blocked functional magnetic resonance imaging (fMRI) experimental design. Twenty-six right-handed participants observed interactions between the right hand and a cup from 1PP and 3PP. The results indicated that both 1PP and 3PP were associated with similar patterns of activation in key areas of the mirror neuron system underlying action observation and understanding. Importantly, besides of the core network of mirror neuron system, we also found that parts of the basal ganglia and limbic system were involved in action observation in both the 1PP and 3PP tasks, including the putamen, insula and hippocampus, providing a more complete understanding of the neural basis for action observation and understanding. Moreover, compared with the 3PP, the 1PP task caused more extensive and stronger activation. In contrast, the opposite comparison revealed that no regions exhibited significantly more activation in the 3PP compared with the 1PP condition. The current results have important implications for understanding the role of the core network underlying the mirror neuron system, as well as parts of the basal ganglia and limbic system, during action observation and understanding.

## Introduction

In the social world, observing, understanding and imitating others’ actions and movements is fundamental to understanding the behaviors, intentions and feelings of others. The neural circuits activated while observing an action performed by another person are similar to the circuits that are activated when one performs the same action (Tkach et al., 2007; Lepage et al., 2008). Such internal simulation of an observed action and the performance of the same action are thought to contribute to action understanding and imitation (Rizzolatti and Craighero, 2004; Gallese, 2009). The study of the neural basis of action observation and intention understanding is not only important for understanding action-related processes, but has broader implications for cognitive and social neuroscience.

Action observation has been proposed to provide an alternative and innovative approach to rehabilitation (Mezzarobba et al., 2017; Scott et al., 2018), and a deeper understanding of the effects of body side, posture, and perspective is crucial for identifying the most effective conditions for stimulation of the motor system during action observation.

The human brain contains a distinct class of neurons called the mirror neuron system (MNS), which discharge both when individuals perform an action, and when they observe another person performing an action with a similar intention (Lago-Rodriguez et al., 2013). It is generally accepted that the MNS plays functional roles in intention understanding (Bonini et al., 2010; Cacioppo et al., 2017), emotion recognition and empathy (Molnar-Szakacs et al., 2009; Goodarzi et al., 2015), action imitation (Molenberghs et al., 2009; Reynolds et al., 2015), self-recognition (Jeon and Lee, 2018) and the evolution of language (Zarr et al., 2013). The MNS transforms sensory information describing the actions of others into a motor representation, which is similar to the representation internally generated by an observer when they actually perform or imagine the action. A direct and automatic matching process between an observed action and past self-related motor experiences allows the observer to understand others’ behavior, as well as their underlying intentions (Fabbri-Destro and Rizzolatti, 2008; Rizzolatti and Fabbri-Destro, 2008; Cacioppo et al., 2017). A longitudinal study showed the appearance and the development of the abilities of children to understanding the intentions of mothers in the first 2 years of life (Capobianco and Cerniglia, 2017; Capobianco et al., 2017), which indicated that gesture-speech combinations play specific roles in children’s early language development. This result is consistent with Liew et al.’s conclusion that the MNS also plays a key role in language-related abilities (Liew and Aziz-Zadeh, 2013).

Observing actions from a first-person perspective (1PP), as if the observer has performed the action themselves, is related to the centrality of the subjective multidimensional and multimodal experience space in one’s own body. This process is constitutive of human self-consciousness (Vogeley and Fink, 2003). In contrast, from a third-person perspective (3PP), an action is observed as if another person is performing it. During the 3PP observation, a mental state is ascribed to someone else. At the underlying representational or cognitive level, these operations need to be clearly distinguished, including the differentiation of different reference frames representing the locations of entities in space (Vogeley et al., 2004). Action observation involves various observer perspectives, whereas the execution of actions only takes place in the “self” reference frame. Stimuli used in the vast majority of studies on action observation have consisted primarily of others’ actions as seen from the 3PP, focusing on what can be seen by an outside observer. During action understanding, visual information from the 3PP needs to translate to the clues and descriptions of motor experiences from the 1PP. Such internal perspective elements of sense are involved in our intentional experience itself, and in the accompanying feeling. Accordingly, the 1PP should also be considered in the interpretation of neurophysiological findings regarding the MNS (Lohmar, 2006). It remains unclear whether action observation and understanding are differentially affected by varying visual perspectives. Assuming that the MNS encodes the intention of an action only by the action goals itself and not the movements to achieve it, in accord with the conventional view (Rizzolatti et al., 2014), the MNS activation pattern would not be expected to be sensitive to the visual perspective. To elucidate this issue, it may be useful to examine action observation and understanding from both the 1PP and 3PP.

Some pioneering studies examined action observation from different visual perspectives. Caggiano et al. (2011) recorded the visual responses of mirror neurons of monkey area F5 to the presentation of grasping motor acts from different visual perspectives. The results revealed that mirror neuron responses were tuned to specific visual perspectives, indicating that the human MNS may also have a directional dependence. Jackson et al. studied the neural circuits involved in the observation of video-clips depicting simple hand or foot actions from the 1PP and 3PP, suggesting that the 1PP is more tightly coupled to the sensory-motor system than the 3PP (Jackson et al., 2006). Macuga et al.’s fMRI study revealed selective responses of the MNS for 1PP and 3PP (Macuga and Frey, 2011). However, both Jackson et al. and Macuga et al.’s studies involved hand or foot actions alone, with no object. Hand-object interactions are frequent and important in human social behavior, while action observation and intention understanding from both the 1PP and 3PP are involved in interactions between people. Thus, the neural activity during the observation of hand-object interactions from the 1PP and 3PP is worthy of further study.

The current study had two main objectives. First, we sought to elucidate the neural basis of action observation and understanding from the 1PP and 3PP. Second, we sought to compare differences in neural activation between the 1PP and 3PP.

## Materials and Methods

### Participants

Twenty-six healthy adults (nine women and 17 men; mean age = 24.08 years, SD = 1.21, range 22–26 years) participated in the study. None of the recruited participants reported neurological or psychiatric histories, or the use of medication. All participants had normal or corrected-to-normal visual acuity, and were right-handed, as confirmed by the Edinburgh Handedness Inventory. The protocol was approved by the Ethics Committee of Affiliated Zhongda Hospital, Southeast University (2018ZDSYLL035-Y01). All participants provided written informed consent in accordance with the World Medical Association Declaration of Helsinki (World Medical Association, 2013) and with the guidelines set for magnetic resonance imaging (MRI) scanning (Simmons and Hakansson, 2011). Each participant received 200 Chinese Yuan for participating in the experiment.

### Stimuli

**FIGURE 1:**
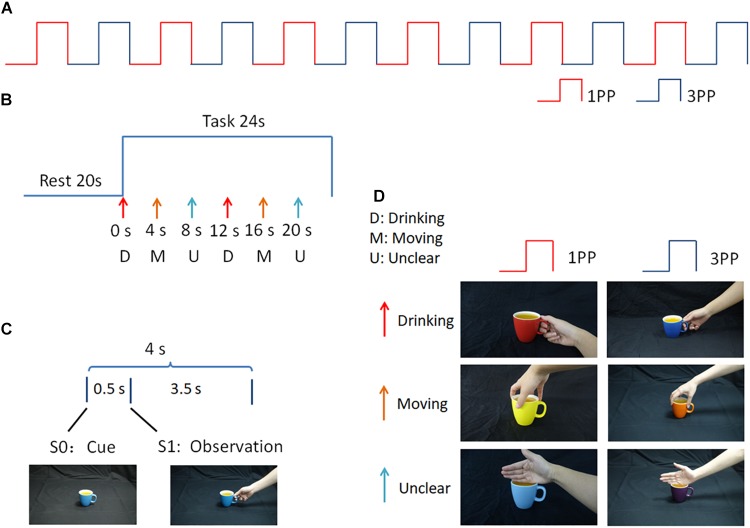
Experimental paradigm. **(A)** 1PP and 3PP stimuli were presented alternately for six blocks; **(B)** Each block consists of rest and task periods. During the task period there were six trials, in which D, M, and U stimuli were displayed sequentially and repeated twice, in different colors; **(C)** Each trial consisted of a hand-cup interaction stimulus presented after a cup-only cue; **(D)** Examples of the stimuli used in this study.

The visual stimuli were programmed in E-Prime (Version 2.0, Psychology Software Tools, Inc., United States) and were divided into two perspective conditions (1PP and 3PP). For each perspective condition, there were three hand-cup interaction conditions corresponding to different underlying intentions, as follows: (a) a right hand grasping the handle of a cup for the purpose of drinking (D), (b) a right hand grasping the rim of a cup for the purpose of moving the cup (M), and (c) a right hand touching the rim of a cup, with an unclear intention (U). To maintain the novelty of the stimuli, in every block, the color of the cup for the six stimuli alternated randomly among seven colors. Examples of the three types of hand-cup interaction under 1PP and 3PP conditions are shown in Figure 1D. The visual stimuli were projected onto a translucent screen located 70 cm away from the participant’s head, via an fMRI stimulation system (SA-9900 Visual & Audio Stimulation System for fMRI, Shenzhen Sinorad Medical Electronics Inc., CHN). A mirror was used to refract the light path so that the participant could see the translucent screen. The size of the stimuli was H: 28 cm (22.62 °) × V: 16 cm (13.04°).

### Procedure

The whole experiment consisted of 12 blocks, in which action observation stimuli under 1PP and 3PP conditions were presented alternately (see Figure 1A). Each block consisted of a 20 s black screen rest period and a 24 s task period. During the task period there were six trials, in which D, M, and U stimuli were displayed sequentially and repeated twice in different colors (see Figure 1B). During each trial, a cue phase lasting 0.5 s, in which a cup appeared without context (S0) as a cue on the screen, prompting the participant to prepare for the hand-cup interaction observation. Then, an observation phase was presented for 3.5 s, in which a hand-on-cup action was presented without context, showing a hand grasping or touching the cup (S1) (see Figure 1C). The gap between the first (S0) and second (S1) stimuli was very short. In this way, the continuous image sequence created the perception of an action (Brown et al., 2010). During the action observation, participants were not required to make a response.

### Data Acquisition and Analysis

All participants were scanned using a GE Discovery MR750 3T magnetic resonance imaging (MRI) whole body scanner (GE Healthcare, United States) with a 32-channel head coil. Displays were presented via a visual and audio stimulation system for fMRI (SA-9900, Shenzhen Sinorad Medical Electronics Co., Ltd., CHN). A trigger pulse from the scanner synchronized the onset of stimulus presentation with the beginning of the image acquisition period.

A single-shot gradient-recalled echo planar imaging (EPI) sequence with sensitivity encoding (SENSE) was employed for blood oxygenation level-dependent (BOLD) functional MRI (fMRI) scans. For an anatomical reference for the fMRI analyses, A T1-weighted imaging was acquired using a 3D-BRAVO sequence. Echo time (TE) = 3.2 ms, repetition time (TR) = 8.2 ms, flip angle = 8°, image matrix = 256 × 256, slice thickness = 1 mm, and field of view (FOV) = 25.6 cm. EPI was performed while subjects carried out the behavioral paradigms using a T2^∗^-weighted gradient-echo EPI pulse sequence. Task state functional BOLD data were acquired using an EPI sequence with FOV = 22 cm, image matrix = 64 × 64, flip angle = 90°, TE = 30 ms, TR = 2000 ms, slice thickness = 3.5 mm, and spacing = 0.5 mm.

First-level fMRI data processing was carried out using FSL (Version 5.0.6, FMRIB Software Library, Oxford, United Kingdom) (Smith et al., 2004; Jenkinson et al., 2012). Before pre-processing, the first two volumes of each voxel’s time course were excluded from analysis to allow the fMRI signal to reach a steady state.

The following data pre-processing procedure was carried out on the fMRI data set using the fMRI Expert Analysis Tool (FEAT, Version 6.0), an embedded module in FSL. A standard pre-processing pipeline was applied, including motion correction using the MCFLIRT method in FSL (Jenkinson and Smith, 2001), non-brain tissue removal using the brain extraction tool (BET) in FSL (Smith, 2002), spatial smoothing using a Gaussian kernel of 5 mm full width at half maximum (FWHM), mean-based intensity normalization of all volumes by the same factor (4D grand demean), and high-pass temporal filtering (100 s). Functional scans were registered to Montreal Neurological Institute (MNI) standard space using affine registration with FLIRT (Jenkinson and Smith, 2001). Time series statistical analysis was performed using FILM in FSL, with local autocorrelation correction (Woolrich et al., 2001).

For individual analysis, a first-level voxel-wise general linear model (GLM) implemented in a FEAT analysis was performed. Z statistic images were thresholded using clusters determined by Z > 2.3 and an adjusted corrected cluster significance threshold of *P* = 0.05. The results were entered into the next higher-level within-group analysis.

For group analysis, a second-level (fixed-effects analysis) cluster-corrected FEAT analysis was performed. All final individual analyses images were thresholded using clusters determined by *Z* > 2.3, and an adjusted corrected cluster significance threshold of *P* = 0.05, corrected for multiple comparisons, was considered significant (Worsley et al., 1992).

In addition, we considered that the sufficient amount and quality of data supporting the results would depend on the degree to which the fMRI BOLD signal fits the experimental design. We investigated the BOLD signal time courses in the IPL and cuneus (see Supplementary Figures S1, S2) and found our fMRI data fits the block design very well.

## Results

### First-Person Perspective

**FIGURE 2:**
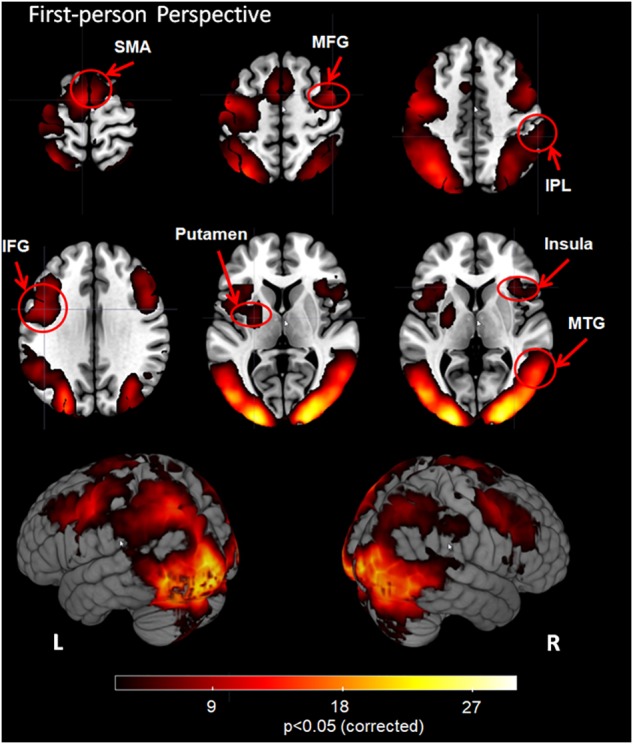
Group analysis results for action observation and understanding from the first-person perspective with a Z-statistic threshold of 2.3 and corrected cluster *P* < 0.05. SMA: supplementary motor area; MFG, middle frontal gyrus; IPL, inferior parietal lobule; IFG, inferior frontal gyrus; MTG, middle temporal gyrus; L, left hemisphere; R, right hemisphere.

The whole-brain fixed-effects group analysis with a Z-statistic threshold of 2.3 (corrected cluster *P* < 0.05) for 1PP revealed significant activation in bilateral visual cortex, supplementary motor area (SMA), middle frontal gyrus (MFG), inferior frontal gyrus (IFG), middle temporal gyrus (MTG), inferior parietal lobule (IPL), insula, putamen and visual cortex including bilateral middle occipital gyrus (MOG), inferior occipital gyrus (IOG) and cuneus (Figure 2).

### Third-Person Perspective

**FIGURE 3:**
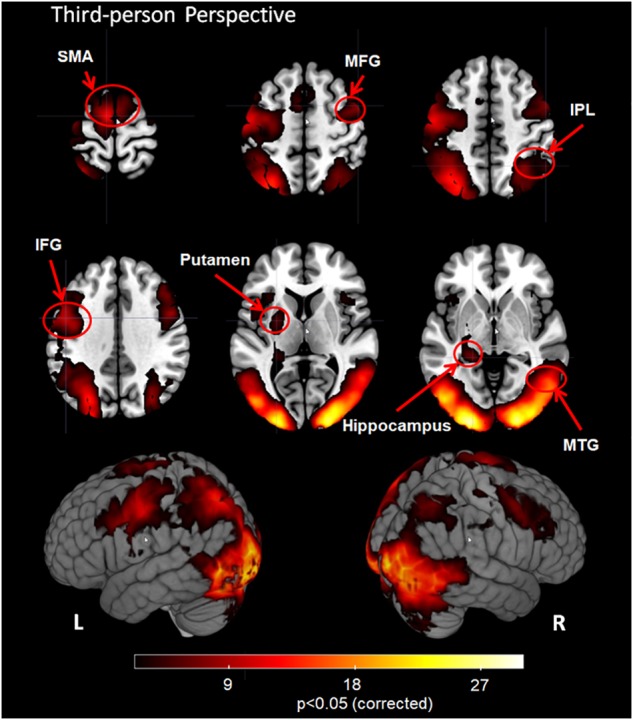
Group analysis results for action observation and understanding from the third-person perspective with a Z-statistic threshold of 2.3 and corrected cluster *P* < 0.05. SMA: supplementary motor area; MFG, middle frontal gyrus; IPL, inferior parietal lobule; IFG, inferior frontal gyrus; MTG, middle temporal gyrus; L, left hemisphere; R, right hemisphere.

The 3PP task induced significant activation in bilateral visual cortex, SMA, MFG, IFG, MTG, IPL, putamen, hippocampus and visual cortex including bilateral MOG, IOG and cuneus (Figure 3, corrected cluster *P* < 0.05).

### First- vs. Third-Person Perspective

**FIGURE 4:**
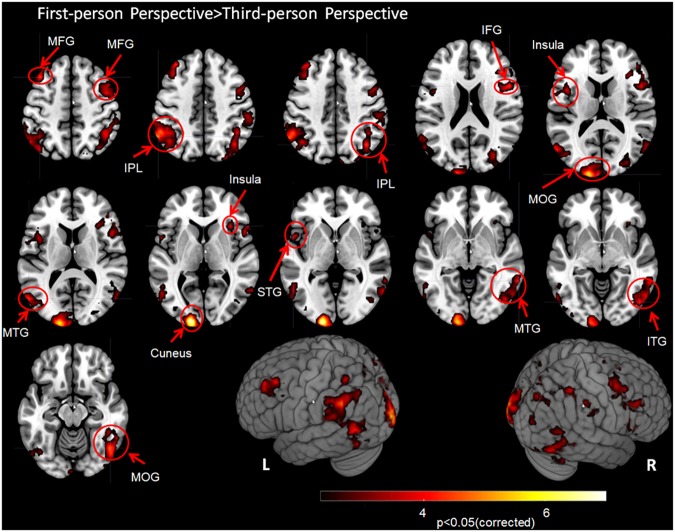
Group analysis results for action observation and understanding from the first-person perspective vs. the third-person perspective with a Z-statistic threshold of 2.3 and corrected cluster *P* < 0.05. MFG, middle frontal gyrus; IPL, inferior parietal lobule; IFG, inferior frontal gyrus; MOG, middle occipital gyrus; MTG, middle temporal gyrus; STG, superior temporal gyrus; ITG, inferior temporal gyrus; L, left hemisphere; R, right hemisphere.

The contrast first- vs. third-person perspective revealed increased activation of the bilateral MTG, bilateral IPL, bilateral insula, right inferior frontal gyrus (rIFG), right middle frontal gyrus (rMFG), right inferior temporal gyrus (rITG), left superior temporal gyrus (lSTG), and visual cortex, including bilateral MOG and left cuneus (Figure 4 and Table 1, corrected cluster *P* < 0.05).

**Table 1:** Activated areas related to the first-person perspective vs. third-person perspective with locations for simple T contrasts.

Cluster Index	L/R	Brodmann area	Regions	Z score	MNI coordinates			Voxels	*P*-Value
					**X**	**Y**	**Z**		
1	L	BA40	inferior parietal lobules	5.00	-58	-56	44	3440	<0.001
1	L	BA39	middle temporal gyrus	4.41	-56	-70	12	^∗^	^∗^
2	L	BA18	cuneus	6.97	-16	-100	4	2607	<0.001
2	L	BA18	middle occipital gyrus	5.94	-16	-102	14	^∗^	^∗^
3	R	BA44	inferior frontal gyrus	4.40	52	10	20	2390	<0.001
3	R	BA6	middle frontal gyrus	3.97	46	2	50	^∗^	^∗^
3	R	BA13	insula	3.66	34	22	4	^∗^	^∗^
4	R	BA7	inferior parietal lobules	4.48	40	-66	42	1865	<0.001
5	R	BA37	middle occipital gyrus	4.69	48	-64	-14	1855	<0.001
5	R	BA21	middle temporal gyrus	4.19	64	-50	-4	^∗^	^∗^
5	R	BA19	inferior temporal gyrus	4.07	48	-74	-6	^∗^	^∗^
6	L	BA8	middle frontal gyrus	4.42	-40	22	50	1252	<0.001
6	L	BA44	insula	3.91	-44	6	14	^∗^	^∗^
6	L	BA22	superior temporal gyrus	3.78	-54	8	0	^∗^	^∗^

*L: Left; R: Right; MNI: Montreal Neurological Institute; ^∗^the peak is in the same cluster as the other peaks. All clusters survived a threshold of *P* < 0.05, corrected for multiple comparisons at the cluster level for the whole brain*.

In contrast, no regions exhibiting more activation were found in the 3PP > 1PP comparison (corrected cluster *P* < 0.05).

## Discussion

In the present study, we used fMRI to investigate the influence of perspective while participants performed motor action observation and understanding from the 1PP and 3PP. The results revealed that the key circuit of the MNS, including the putamen, insula and hippocampus, was involved in action observation and understanding from both the 1PP and 3PP. Most previous neuroimaging studies of action observation and understanding focused on the MNS, including the SMA (Grezes and Decety, 2001; Cross et al., 2009; Balser et al., 2014; Li et al., 2015), MFG (Caspers et al., 2010; Plata et al., 2014), IFG (Grezes and Decety, 2001; Caspers et al., 2010), MTG (Tamura et al., 2013), IPL (Balser et al., 2014; Plata et al., 2014), putamen (Willems and Hagoort, 2009; Jelsone-Swain et al., 2015), and insula (Caspers et al., 2010; Plata et al., 2014). The current results are in accord with these previous observations.

The SMA is thought to be sensitive to self-integration during observation (Zentgraf et al., 2005). One previous study suggested that the SMA may be related to subjective experience of mental imagery, which participants engage in during the observation of familiar action sequences (Cross et al., 2009). In addition, this region is known to be activated by motor imagery (Park et al., 2015) and action simulation (Grezes and Decety, 2001). Observing action has been found to lead to internal execution (action simulation) (Zentgraf et al., 2005). It is possible that the SMA is triggered by the transformation of the visual template into body-centered coordinates (Zentgraf et al., 2005).

Fogassi et al. (2005) reported that the IPL discriminates identical motor acts according to the action in which these acts are embedded. Creem-Regehr (2009) proposed that the IPL underlies motor cognition, which includes the generation of internal representations of actions. This region is also considered crucial for the use of objects and tools, object-related action schemata, gestural praxis, and action manipulation (Rumiati et al., 2004; Buxbaum et al., 2005), which help to generate internal models of hand-object interaction.

During both action observation and imitation, as a classical MNS region, the IFG is thought to be involved in self-recognition (Macuga and Frey, 2011), subsequent action prediction (Iacoboni et al., 2005) and tool use associated with motor control (Tamada et al., 1999). Mirror neurons in the IFG have also been suggested to encode concrete/pragmatic representations (Kilner, 2011; Press et al., 2012) and the goal of an observed action (Gallese et al., 1996). The MFG also plays an important role in motor processing related to imitation (Sugata et al., 2017).

An fMRI study by Jastorff et al. (2010) revealed that the MTG is sensitive to the rationality of an action and, as the authors noted, the MTG is located in close proximity to the superior temporal sulcus (STS), which is a “core circuit” for action observation (Perrett et al., 2009; Biagi et al., 2010). The MTG/STS regions do not only analyze the kinematics of body and body movements, but also combine these biological movement cues with the relevant context in a visual stimulus (Jastorff et al., 2010).

Action observation may reflect the widespread influence of the prefrontal cortex and ventral pathways (Arbib, 2010), whereas the STS codes visual perception, the MTG is responsible for the sensory representation (Gazzola and Keysers, 2009), and the IFG is responsible for coding the goal of the action (Gallese et al., 1996). Schubotz et al. (2014) suggested that the MTG and IPL both co-varied in activation during action coding, because action coding differs not only with regard to the way we use objects, but also in relation to the way objects move when they are used. Kilner suggested that the link between the MTG and IFG form a ventral pathway, which generates a gradient of the representation of the action from the semantic level, through to the goal level, to the concrete level (Kilner, 2011).

Some action recognition models assume a hierarchy of processing steps, indicating that the visual details of observed actions are mainly processed in higher-order visual information processing areas, including the MTG, and STS (Oztop and Arbib, 2002; Bonaiuto and Arbib, 2010). In contrast, the MNS achieves action understanding by matching the visual and motor representations of the observed actions. This mechanism is considered to be the neural basis of understanding the behavior and intentions of others actions (Cook et al., 2014).

Some previous MNS studies have focused on the observation perspective. A study of mirror neurons in monkey area F5 (corresponding to the human MNS) reported that the majority of tested mirror neurons exhibited perspective-dependent activity with responses tuned to a specific perspective (Caggiano et al., 2011). The researchers proposed that although view invariance is processed in higher-order visual areas (e.g., the STS), this processing is not complete or sufficient. These findings suggest that the MNS plays an essential role in the formation of view-invariant representations. It is plausible that the MNS contributes to the modulation of perspective-dependent representations coming from the higher-order visual areas, potentially in trials of action goals/intentions and independent of their detailed visual aspects.

Interestingly, we found that parts of the basal ganglia and limbic system were involved in action observation, including the putamen, insula and hippocampus, in both the 1PP and 3PP tasks. Compared with the above-mentioned core network of the MNS, there have been fewer reports of putamen activation during action observation/understanding tasks. Previous studies have reported that the putamen is associated with automatic movement behaviors (Ashby and Crossley, 2012), habitual control of behavior (Balleine and O’Doherty, 2010), motor imagery (Taube et al., 2015), execution of movement (Ueda and Kimura, 2003), and visuomotor information processing (Romero et al., 2008; Vicente et al., 2012). Hand movement observation has also been reported to cause activation in the putamen (Willems and Hagoort, 2009). Some researchers have proposed that putamen activity is correlated with implicit and reinforcement learning for movement (Packard and Knowlton, 2002; Vicente et al., 2012). Yin et al. found putamen activation during movement sequences (Yin and Knowlton, 2006; Marchand et al., 2008), and Balleine et al. reported that the putamen is activated when the predicted outcome of an action is the same as the outcome when the action is performed (Balleine and O’Doherty, 2010). A study by Rubichi et al. (2011) suggested that action observation causes implicit learning. Taken together with the current results, the above findings suggest that the putamen is involved in action observation, in which visual information (observed action) is combined with internal similar action sequences (automatic/habitual behavior) via implicit/reinforcement learning and imitation. In this process, the observer would be expected to perform an action internally that is similar to the action they observed, enabling the observer to understand action intention via action observation.

The insular regions may be linked to bodily self-consciousness (Tsakiris et al., 2007), intentional actions (Brass and Haggard, 2010), observation and imitation of facial expressions (Carr et al., 2003), and empathy for experiences involving pain (de Vignemont and Singer, 2006) and disgust (Wicker et al., 2003). These findings indicate that the insula may be associated with a mirror neuron-like link between external and internal experiences, enabling the observer to recognize the observed action. In addition, previous research also suggested that the insula may play a key role in the experience of the self or others as the cause of an action (Farrer and Frith, 2002), and self-perception during observation action of the self or others (Macuga and Frey, 2011). Such findings indicate that the insula may be involved in the perception of complex representations of an action itself, and the experience of the sense of agency (the cause of an action) during action observation from different perspectives.

Mukamel et al. reported that the hippocampus responded to both action observation and execution (Frey and Gerry, 2006; Mukamel et al., 2010). A previous study of facial expression observation also indicated that besides the “core network” of the MNS, there was also activation in the hippocampus (Likowski et al., 2012). In addition, another study reported that observing the regrettable outcomes of others’ choices activates the hippocampus (Canessa et al., 2009). Researchers have also reported that the hippocampus is critical for processing temporally-structured information and associated events that are separated in time and/or space (Schendan et al., 2003). Therefore, we considered that, during action observation, the hippocampus may play a role in combining sequential events into a unique episodic experience, consistent with relational or similar movement memory. Such processing of recall memory may help to understand the observed action.

Watanabe et al. (2013) performed an action imitation fMRI study, revealing that imitation based on the 1PP was associated with significantly stronger brain activation than that in the 3PP condition, whereas no brain regions were significantly more strongly activated in the 3PP compared with the 1PP condition. In the current study, we also found more significant activation in the 1PP than in the 3PP condition, particularly in the fronto-parietal MNS network and visual cortex. In contrast, no brain regions showed significantly more activation in the 3PP compared with the 1PP. These results are in accord with Watanabe et al.’s findings (Watanabe et al., 2013). Jackson et al. (2006) suggested that the 1PP is more extensively coupled to the sensory-motor system compared with the 3PP, regardless of whether the movements are produced or not. In the current study, stronger brain activity in the core MNS circuit was observed when participants observed the actions from the 1PP compared with the 3PP, which is consistent with the studies described above (Jackson et al., 2006; Watanabe et al., 2013). Similar to the view of Jackson et al. (2006), we considered that action observation and understanding from 1PP is congruent with the “self” perspective, while the 3PP is closely related to the “others” perspective. The current results suggest that regardless of the underlying intention of actions, observation of actions from the 1PP induced more involvement in the core MNS circuit than the 3PP, which may induce neural activity reflecting the tendency for observed actions from the 1PP to be processed as “like me,” activating self-action (Meltzoff and Decety, 2003; Meltzoff, 2005).

## Conclusion

The current study provides a preliminary step in the neuroscientific investigation of the neural correlates of action observation and understanding from 1PP and 3PP. Based on the current findings, we suggest that the neuronal activation patterns underlying action observation and understanding from these two perspectives similarly involve the main parts of the MNS. Besides the core network of MNS, the current study revealed activation in the putamen, insula, and hippocampus, which indicated that the implicit/reinforcement learning and imitation, the experience of the sense of agency, and the recall of movement memory may also be involved in action observation and understanding. In addition, we found significantly more extensive and stronger activation in the 1PP compared with the 3PP. In contrast, we observed no regions that showed significantly more activation in the 3PP compared with the 1PP. These findings enable a more complete understanding of the neural basis of action observation and understanding.

## Author Contributions

SG conducted the experiments, data analysis, and wrote the manuscript. HL, PL, and JG conducted the experiments and data analysis. CX and ZL conducted the fMRI experimental paradigm design.

## Conflict of Interest Statement

The authors declare that the research was conducted in the absence of any commercial or financial relationships that could be construed as a potential conflict of interest.
