# Supplementary material for: Neural Basis of Action Observation and Understanding From First- and Third-Person Perspectives: An fMRI Study
Source: Front Behav Neurosci. 2018 Nov 22;12:283. doi: 10.3389/fnbeh.2018.00283 (PMC6262037; doi:10.3389/fnbeh.2018.00283)
Supplement: Supplementary file 1 [file Data_Sheet_1.docx]

Supplementary Material

Neural Basis of Action Observation and Understanding from First- and Third-person Perspectives: An fMRI Study

Sheng Ge, Hui Liu, Pan Lin, Junfeng Gao, Chaoyong Xiao, Zonghong Li^*^

*** Correspondence:** Zonghong Li: 13951734989@163.com

## Supplementary Figures


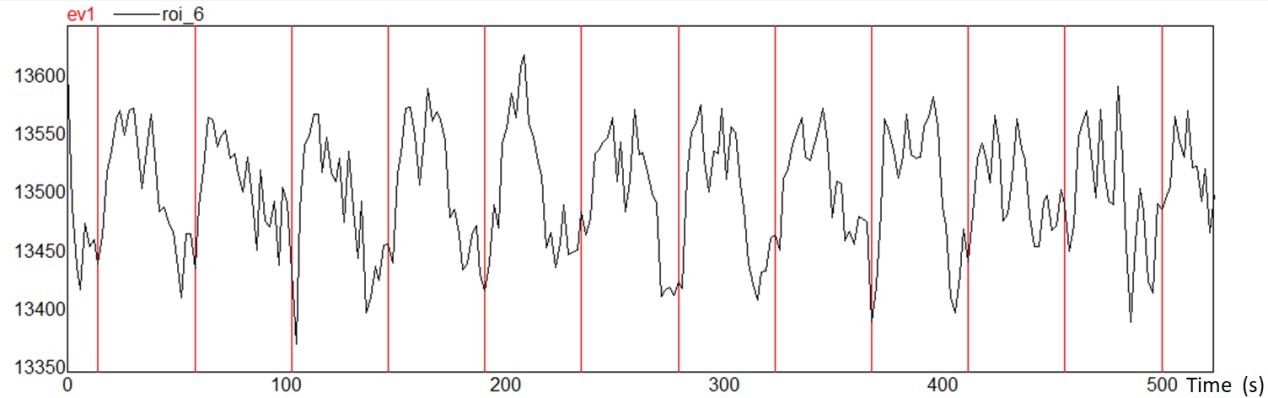


**Supplementary Figure 1.** BOLD signal time course for IPL.


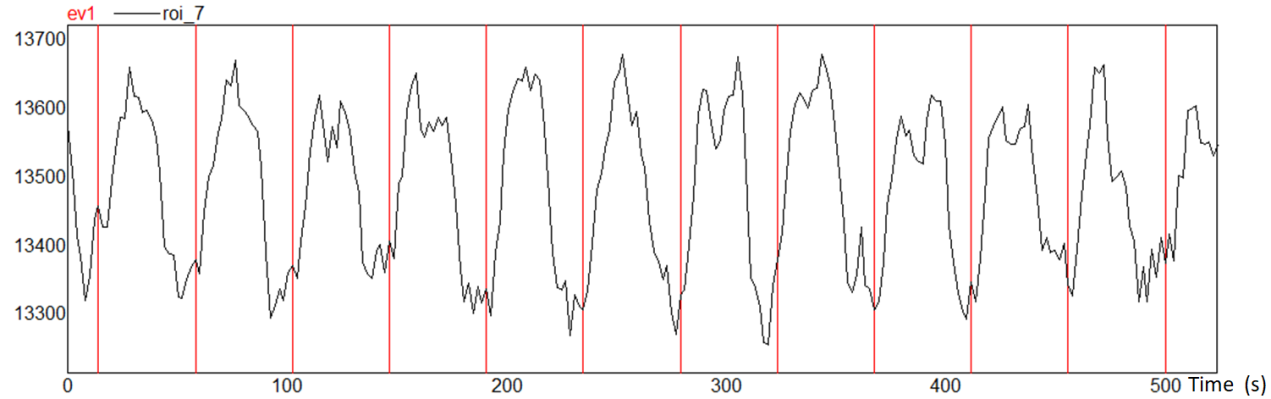


**Supplementary Figure 2.** BOLD signal time course for cuneus.
